# Emergent Conversion to Open Heart Surgery during Transcatheter Aortic Valve Implantation: The Presence of a Rescue Team Improves Outcomes

**DOI:** 10.3390/jcm12247705

**Published:** 2023-12-15

**Authors:** Giuseppe Nasso, Walter Vignaroli, Gaetano Contegiacomo, Alfredo Marchese, Khalil Fattouch, Pasquale D’Alessandro, Mario Siro Brigiani, Vincenza Vitobello, Vera Triggiani, Maria Antonietta Demola, Stefano Tonioni, Domenico Paparella, Stefano Sechi, Raffaele Bonifazi, Giuseppe Santarpino, Fabrizio Resta, Francesco Bartolomucci, Roberto Lorusso, Claudio Larosa, Giovanni Valenti, Antonio Tito, Marco Moscarelli, Vito Margari, Flavio Fiore, Ignazio Condello, Giuseppe Speziale

**Affiliations:** 1 Department of Cardiac Surgery, Anthea Hospital, GVM Care & Research, 70124 Bari, Italy; vignaroli.walter@gmail.com (W.V.); gcontegiacomo@gvmnet.it (G.C.); pasdalessandro@libero.it (P.D.); msirobrigiani@gvmnet.it (M.S.B.); vincenzavitobello@gmail.com (V.V.); veratriggiani@libero.it (V.T.); mariaantoniettademola@gmail.com (M.A.D.); bgiava@tiscali.it (R.B.); gsantarpino@gvmnet.it (G.S.); mmoscarelli@gvmnet.it (M.M.); ffiore@gvmnet.it (F.F.); ignicondello@hotmail.it (I.C.); gspeziale@gvmnet.it (G.S.); 2 Department of Cardiac Surgery, San Carlo di Nancy, GVM Care & Research, 00165 Roma, Italy; khalilfattouch@hotmail.com (K.F.); ste.ton@tiscali.it (S.T.); s.sechi88@gmail.com (S.S.); 3 Department of Cardiac Surgery, Santa Maria Hospital, GVM Care & Research, 70124 Bari, Italy; alma@libero.it (A.M.); fabrizioresta@gmail.com (F.R.); anto.tito@libero.it (A.T.); vitomargari@icloud.com (V.M.); 4 Department of Cardiology, Hospital of Andria, 76123 Andria, Italy; f.bartolomucci@aslbat.it (F.B.); larosa.cld@gmail.com (C.L.); gv1269@gmail.com (G.V.); 5 Cardio-Thoracic Surgery Department, Heart and Vascular Centre, Maastricht University Medical Centre (MUMC), 6229 HX Maastricht, The Netherlands; roberto.lorusso@mumc.nl

**Keywords:** TAVI, cardiac surgery, heart team, conversion to open heart surgery

## Abstract

Objective: Transcatheter aortic valve implant (TAVI) is the gold standard for the high-surgical-risk group of patients with aortic valve disease and it is an alternative to surgery in patients at intermediate risk. Lethal complications can occur, and many of these are manageable only with emergent conversion to open heart surgery. We retrospectively evaluate the outcome of all patients undergoing TAVI in our departments and the impact of a complete cardiac rescue team to reduce 30-day mortality. Methods: Data from all patients undergoing TAVI between January 2020 and August 2023 in our center were analyzed. An expert complete rescue was present in catheter laboratory. Primary outcomes were in-hospital and at 30-day mortality and evaluation of all cases needed for emergent conversion to open heart surgery. Results: 825 patients were enrolled. The total mortality was 19/825 (2.3%). Eleven of the total patients (1.3%) required emergent conversion to open heart surgery. Among them, eight were alive (73%), with a theoretical decrease of 0.98% in overall mortality. Conclusions: surgical treatment is rare during TAVI. The presence of an expert complete rescue team as support means an increase in survival. Surgery must be used only to restore circulatory and to treat complication while percutaneous approaches should complete the procedure.

## 1. Introduction

Aortic valve stenosis is the most common valvular pathology, with 2–5% of the population over 65 years old affected [1]. Surgical aortic valve replacement (SAVR) is the class I° recommendation for managing symptomatic aortic stenosis. However, transcatheter aortic valve implant (TAVI) has emerged as the therapeutic gold standard for a high-surgical-risk group of patients [2]. Thanks to several trials, TAVI is alternative to SAVR in patients at intermediate surgical risk. An increasing number of patients with low surgical risk are now candidates for this procedure [3,4] and as a consequence, today, TAVI is alternative to SAVR for patients across all risk groups.

According to the United States Aortic Valve Registry, already in the calendar year 2019, TAVI surpassed 70,000 implants and compared with SAVR the ratio is 2:1.

TAVI is associated with a higher incidence rate of permanent pacemaker implantation (12.5%) [5], paravalvular leak (7–33%) [6], and vascular complications (9.5–15%) [7] while SAVR results in higher rates of bleeding complications (10%) [8], acute kidney injury (26.3%) [9] and arrhythmias (20–35%) [10].

The transfemoral approach is the most used (70%), but alternative approaches are transapical, transaxillary, transcarotid, and transaortic, with optimal clinical results [11].

European and American guidelines on aortic valve disease recommend performing TAVI in ‘Heart Valve Centers’ with interventional cardiology and institutional on-site Cardiac Surgery (iOSCS) with 24 h/7-day services [12,13]. A hybrid catheterization laboratory is desirable but not essential. Actually, guidelines do not approve TAVI in centers without iOSCS.

Even if TAVI procedures have become safer thanks to increasing operator experience and technological developments in devices, lethal complications can still occur, many of them manageable only with emergent conversion to open heart surgery (E-OHS) [14,15].

The association with the continuous growth of candidates for transcatheter interventions leads to increased waiting times for procedures with negative consequences on mortality, morbidity, repeated hospitalizations, and functional deterioration; therefore, several trials are ongoing to evaluate the results of TAVI in centers without cardiac surgery on site [16].

Studies exclusively focused on surgical E-OHS are few and with low number of patients. Furthermore, considering that the techniques are constantly evolving, the results are in many cases based on old methods [15,17,18].

This article evaluated the results of all patients undergoing TAVI in our departments and the consequence of a complete and ace rescue team presence with cardiac surgeon, surgery nurse and perfusionist, during emergency, to reduce 30-day mortality. The best treatment during emergent cardiac E-OHS in additionally analyzed.

## 2. Materials & Methods

### 2.1. Study Population

From January 2020 to August 2023, a total of 825 consecutive patients with aortic valve stenosis who underwent to TAVI were evaluated at the cardiac departments of “Anthea Hospital” GVM Care & Research—Bari, “Santa Maria Hospital” GVM Care & Research—Bari, and “San Carlo di Nancy” GVM Care & Research—Roma, Italy.

According to the current guidelines, the heart team met collectively and directed the patients to TAVI because of their advanced age or because conventional surgery to replace the aortic valve was associated with high or intermediate surgical risk, according to EUROSCORE II, or in the case of low risk but with a particular condition unfavorable for SAVR.

All clinical data were retrospectively extrapolated by our general and cumulative registry database (containing clinical information of all patients admitted to the hospitals) and then retrospective analyzed. The study conforms to the ethical principles of the Good Clinical Practice, the Helsinki Declaration, and is in compliance with the current regulations.

All patients gave written informed consent for inclusion, collection/use of data or samples, and/or publication according to the actual guidelines. IRB number for the research protocol is TB001.

Baseline characteristics were defined according to the European System for Cardiac Operative Risk Evaluation (EuroSCORE II) definitions and are summarized in Table 1.

Of 825 patients, 396 were females (48%), mean age was 79 years ± 6.4. 779 patients (94.4%) had an elective indication, and the remaining 46 (5.6%) had an urgent/emergent approach. The average EUROSCORE II was 7 ± 2. 46 patients (5.6%) had bicuspid valve (all type 1 of Sievers classification [19]). A total of 149 patients (18%) were previously submitted to cardiac surgery and 64 (7.8%) of those had a previous surgical aortic valve replacement.

### 2.2. Surgical Procedure

The procedure was performed with conscious sedation and local anesthesia. Initially, femoral venous access was obtained, and a temporary pacing wire was positioned in the right ventricle. A 6-Fr (French) 25 cm arterial sheath was inserted in the left radial artery, and a selective left/right coronary angiography was performed; when necessary, we proceeded to PCI (percutaneous coronary intervention) in the same session. Fluoro-guided puncture of the main access was performed, injecting through a 6-Fr MPA1 (Multipurpose Amplatz 1) catheter in the ipsilateral common iliac artery via the left radial route. The 6-Fr 10 cm femoral arterial sheath was positioned, and 1 or 2 suture-mediated closure devices (Perclose ProGlide^®^, Abbott Laboratories, Abbott Park, IL, USA) were pre-implanted, depending on the sheath of the prosthesis to be implanted (14F or 16F, respectively). Intravenous unfractionated heparin was administered to achieve an activated clotting time (ACT) > 250 s. The 6-Fr 10 cm femoral arterial sheath was exchanged with a 10-F 10 cm introducer. For angiographic checks, the pigtail catheter was passed through the left radial sheath and positioned in the basal portion of the non-coronary cusp. The native valve was crossed using a 300 cm straight-tip wire, advanced with AL1 (Amplatz Left 1) catheter, exchanged with a 0.035 inch pre-shaped 300 cm stiff guidewire (INNOWI^®^, SYMEDRIX GmbH, Oberhaching, Germany) positioned in the left ventricular apex. A pre-implantation balloon aortic valvuloplasty was usually performed with semi-compliant expansion devices (VACS-II balloons, Osypka AG, Rheinfelden, Germany) through the 10-Fr femoral arterial sheath, with rapid right ventricular pacing; the balloon size was based on the minor diameter of the aortic annulus. The delivery system of the prosthesis was inserted in the main access after removal of the 10-Fr introducer and proceeded with angio- and fluoroscopic-guided valve implantation. After release, a final angiogram and a subsequent transthoracic echocardiographic check were performed to assess valve placement and significant paravalvular leaks to exclude interference with the mitral valve and any signs of new onset pericardial effusion. If the implant was deemed satisfactory and it was unnecessary to proceed with post-dilations, the large sheath delivery system was removed over a J-tipped 300-cm guidewire. Hemostasis was obtained using pre-implanted suture-mediated closure devices (Perclose ProGlide^®^, Abbott Laboratories, Abbott Park, IL, USA), with the possible use of additional closure devices (Angio-Seal™, Terumo Interventional Systems, Terumo Medical Corporation, Somerset, NJ, USA) or compressive dressings, in case of ineffective hemostasis. Protamine e.v. is administered. At the end of the case, angiographic control of the main access was performed using an MPA1 catheter from the left radial route. The need to keep the temporary stimulator in place or remove was assessed. In case of removal, hemostasis is achieved by manual compression.

The procedure was always performed in a catheter laboratory with an expert intervention cardiologist and an expert cardiac surgeon as support. A complete stand-by cardiac surgery equipped and accustomed to emergency management for E-OHS was always present during the procedure.

An anesthesiologist is always present during TAVI procedure and during E-OHS the procedures were all converted to general anesthesia with orotracheal intubation.

The E-OHS is always performed with an immediate sternotomy and direct hear resuscitation in cases without history of cardiac surgery. In cases with previous cardiac surgery an ECMO is positioned peripherally and then re-sternotomy is performed. We take a maximum of 20 min to guarantee circulation support in both cases.

**Figure 1:**
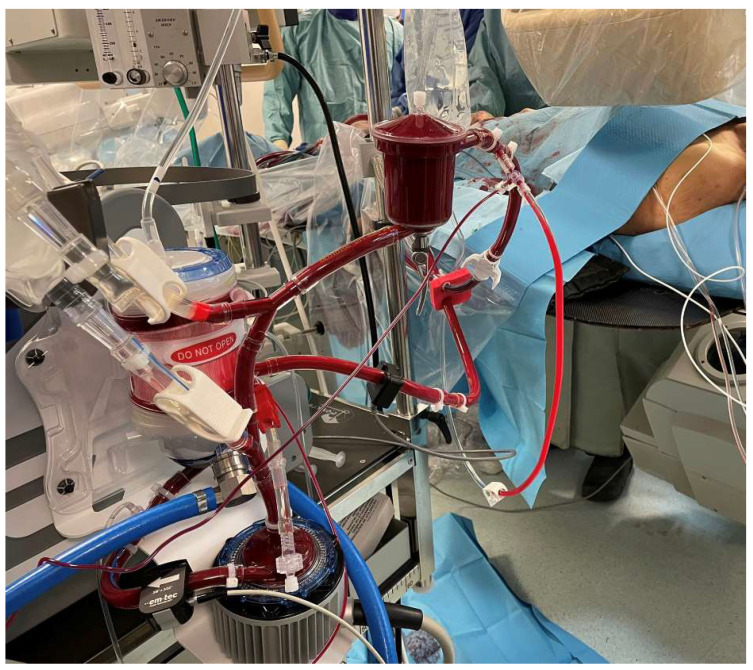
ECMO setup on surgical room used during the procedure.

The ECMO setup is exposed in Figure 1.

### 2.3. Study Endpoint

The patient’s post-operative status was monitored via outpatient clinic visits and telephone interviews.

Two primary study endpoints are defined:

Overall mortality or major complication within 30 days of TAVI procedure.

Evaluation of all cases that needed an E-OHS during the procedure with the consequent impact on overall mortality.

### 2.4. Statistical Analysis

Data are expressed as mean + standard deviation for continuous variables and as a percentage for categorical variables. Continuous variables were compared using the paired *t*-test with a significance level (*p*-value < 0.05), while categorical variables are presented as frequencies and percentage.

## 3. Results

### 3.1. Operative Outcomes

We used 4 types of aortic prostheses: in 448 cases (54.3%), “Corevalve Evolute R” (Medtronic, Minneapolis, MN, USA), in 281 (34.1%) cases, a “Portico” (Abbott Vascular, Santa Clara, CA, USA), in 94 cases (11.4%) a “Corevalve” (Medtronic, Minneapolis, MN, USA) and in 2 cases (0.2%) a “Myval” (Meril Life Sciences Pvt. Ltd., Vapi, Gujarat, India).

The average dimension of the prosthesis was 27.5 mm. The most used dimensions were 26 mm (120 cases—21%), 27 mm (71 cases—12.5%), and 29 mm (159 cases—28%).

A total of 42 patients (5.1%) were also treated by PCI immediately before valve implantation for concomitant significative coronary artery disease.

The trans-femoral approach was not feasible in 12 patients (1.4%). Therefore, we performed 6 trans-subclavian TAVI (0.7%) with surgical artery exposure and, in the following cases, 6 trans-axillary TAVI (0.7%) with a percutaneous approach.

Thirteen cases (1.6%) developed cardiogenic shock, 2 were treated with pharmacological support, and 11 with E-OHS.

Two patients with post-procedural complications had been excluded from this group because the surgical approach was not in emergency: in the first case the ventricular damage was very sneaky, and the cardiac tamponade occurred 24 h later. The second case developed a severe mitral regurgitation related to a systolic anterior movement one week after the procedure.

Of 11 patients (1.3%) who needed E-OHS with median sternotomy and cardiopulmonary bypass for cardiogenic shock followed by immediate cardiac arrest (CA), 8 were alive (73%).

All cases with CA treated by opening the chest and cardiopulmonary bypass 8 had a major cardiac perforation; in 1 patient the left main closure caused the CA, 1 developed a circumflex coronary artery occlusion subsequence of a ventricular perforation and 1 had an aortic dissection.

In-hospital mortality was 19 out of 825 patients (2.3%). No adjunctive mortality was detected at 30 days follow-up.

We also had 10 patients (1.2%) with major vascular complications and 26 minor (3.2%), which we resolved with a surgical approach for hemostasis at the end of the procedure.

A total of 58 patients (7%) needed definitive pacemaker implantation.

The in-hospital stay was 10.45 ± 3 days (Table 2).

### 3.2. Analysis of 11 E-OHS Cases

Data of this subgroup of patients, according to VARC 3 criteria [20], are summarized in Table 3.

Eight patients survived at 30 days follow-up. One died during the cardiac surgery, one died on the first post-operative day, and another on fifth post-operative day.

Case 1: 85-year-old man, no previous cardiac surgery. EuroSCORE 5.03%, ejection fraction (EF) 45%, diabetes, arterial hypertension, and mild renal failure. Clinical frailty scale (CFS) 4. We performed trans-axillary approach due to calcified femoral arteries and abdominal aorta extremely tortuous. Due to perforation, he underwent cardiogenic shock and CA during the procedure’s early stages after positioning a stiff guidewire. Opening the sternum and using CPB to support the TAVI release was pivotal. We closed a breach in the left ventricle without the aortic cross-clamp. After that, the TAVI procedure was completed with a Portico n° 25 prosthesis implantation. The patient survived the procedure and was discharged home 18 days later (10 long of stay (LOS) in ICU). At 30 days follow-up, he was still alive. The summary of the hospitalization highlighted the presence of a mild paravalvular leak, and the need for three units of blood transfusion.

Case 2: 84-year-old man, EuroSCORE 4.88%, EF 50%, arterial hypertension, diabetes, dyslipidemia, mild renal failure, and COPD. CFS 5. He underwent cardiogenic shock and CA immediately after the valve release. Valve pop-out and aortic dissection were detected. We performed a sternotomy after peripheral cannulation for CPB. A “Bentall-De Bono” procedure was performed for ascending aortic plus aortic valve replacement. The patient died on the fifth post-operative day due to multi-organ failure.

Case 3: 70-year-old woman, previous cardiac surgery. EuroSCORE 5.18%, EF 60%, arterial hypertension, and dyslipidemia. CFS 3. She underwent cardiogenic shock and CA immediately after the valve release with echocardiography evidence of cardiac tamponade due to apex perforation. After the re-sternotomy, we performed CPB, drained the pericardial effusion, and closed the apex breach without an aortic cross-clamp. The patient survived the procedure and was discharged home 11 days after the procedure (LOS 6). At 30 days follow-up, the patient was alive. The summary of the hospitalization highlighted the need for definitive biventricular pacemaker implantation due to a third-degree AV block.

Case 4: 65-year-old woman. EuroSCORE 5.98%, EF 40%, arterial hypertension, dyslipidemia, lower limb arterial stenosis, COPD, and diabetes. CFS 4. She went into cardiogenic shock immediately after we used a 20 Fr balloon to dilate the aortic valve with echocardiography evidence of cardiac tamponade. The correct diagnosis is established by echocardiography, aortography, coronary angiography, and clinically by direct exploration through a median sternotomy. We found a 1 cm gap on the left ventricular surface, which was 12 mm away from the left ventricle’s lateral wall in proximity to the distal coronary circumflex artery. Due to the impossibility of mobilizing the heart, we performed cardioplegic arrest with cross-clamping and closed the breach. After protamine administration, the interventional cardiologist completed the TAVI procedure. The patient survived the procedure and was discharged home 9 days after the procedure (LOS 5). At 30 days follow-up, the patient was still alive. The summary of the hospitalization highlighted the need for two units of blood transfusion [21].

Case 5: 82-year-old woman, EuroSCORE 4.1%, EF 55%, arterial hypertension, and dyslipidemia. CFS 4. She underwent cardiogenic shock immediately after the valve release with echocardiography evidence of cardiac tamponade. Deepening the diagnosis, a ventricle rupture was evident with massive mitral regurgitation. We performed sternotomy, CPB, and the patient underwent emergency surgery for ventricle repair without an aortic cross-clamp. After surgery, a massive mitral regurgitation was still evident, so a coronarography and a complete occlusion of terminal circumflex coronary artery and of an obtuse marginal were performed. The attempt at PCI was ineffective, and the patient died immediately.

Case 6: 76-year-old man, EuroSCORE 7.03%, EF 40%, arterial hypertension, dyslipidemia, diabetes, severe carotid and lower limbs stenosis, and atrial fibrillation with a history of PCI. CFS 4. He went into cardiogenic shock and CA immediately after the valve release, with angiography evidence of occlusion of the left main coronary artery. We performed sternotomy and CPB for resuscitation and heart support during PCI without an aortic cross-clamp. PCI rescue of left main was successfully done with three stents implanted. The patient survived the procedure and was discharged home 30 days after the procedure (LOS 24). The summary of the hospitalization highlighted the presence of a mild-moderate paravalvular leak, acute kidney injury requiring temporary renal replacement therapy, and the need for two units of blood transfusion.

Case 7: 78-year-old woman, EuroSCORE 4.46%, EF 45%, arterial hypertension, diabetes, and dyslipidemia. CFS 3. She underwent cardiogenic shock and CA immediately after the valve release with echocardiography evidence of cardiac tamponade. We performed a sternotomy to drain pericardial effusion, but we also needed CPB to solve the cardiogenic shock without an aortic cross-clamp and to close the cardiac perforation. The patient survived the procedure and was discharged home 15 days after the procedure (LOS 8). At the 30-day follow-up, the patient was alive. The summary of the hospitalization highlighted the need for one unit of blood transfusion.

Case 8: 82-year-old man, EuroSCORE 1.7%, EF 50%, recent surgery for colon cancer. CFS 5. He underwent cardiogenic shock immediately after the valve release with echocardiography evidence of cardiac tamponade due to free wall left ventricle perforation. Firstly, we performed a pericardiocentesis then, due to continuous drainage replenishment, we opted for sternotomy. We drained the pericardial effusion and closed the breach without an aortic cross-clamp. The patient survived the procedure and was discharged 7 days after the procedure (LOS 3). At the 30-day follow-up, the patient was alive. The summary of the hospitalization highlighted an episode of one day delirium without neurological injury.

Case 9: 82-year-old woman, EuroSCORE 3.35%, EF 55%, arterial hypertension, pulmonary hypertension, chronic lung disease, dyslipidemia, severe neurological disease (a consequence of cerebral ictus), and severe carotid stenosis. CFS 4. She underwent cardiogenic shock and CA immediately after balloon pre-dilatation with echocardiography evidence of cardiac tamponade. We performed CPB to resuscitate and solve the tamponade. After stabilization of hemodynamic parameters, we deepened the diagnosis with evidence of a rupture of the mitro-aortic junction. Through aortic cross-clamp, we repaired the mitro-aortic junction and replaced the aortic valve. Unfortunately, the patient died the next day due to severe heart failure.

Case 10: 78-year-old woman, previous cardiac surgery. EuroSCORE 4.1%, EF 60%, arterial hypertension, and atrial fibrillation. CFS 4. She underwent cardiogenic shock and CA immediately after the valve release with echocardiography evidence of cardiac tamponade due to the anterior wall of the left ventricle perforation. After sternotomy, we performed CPB and drained the pericardial effusion; we also closed the breach without an aortic cross-clamp. The patient survived the procedure and was discharged home 7 days after the procedure (LOS 2). At the 30-day follow-up, the patient was alive. The summary of the hospitalization highlighted the need for one unit of blood transfusion.

Case 11: 83-year-old woman. EuroSCORE 8.3%, EF 45%, arterial hypertension, dyslipidemia, atrial fibrillation, and diabetes. CFS 5. She went into cardiogenic shock and CA immediately while removing wire with echocardiography evidence of cardiac tamponade due to the lateral wall of the left ventricle perforation. After sternotomy, we performed CPB and drained the pericardial effusion; we also closed the lateral breach without the aortic cross-clamp. The patient survived the procedure and was discharged home 20 days after the procedure (LOS 15). At the 30-day follow-up, the patient was alive. The summary of the hospitalization highlighted an episode of transient ischemic attack without neurological evidence of neural damage, acute kidney injury with permanent renal therapy replacement and need for definitive biventricular pacemaker implantation due to a third-degree AV block, and the need for one unit of blood transfusion.

The mean EuroSCORE of patients with E-OHS was 4.91 ± 1.77. 7 was female (63%). Two (18%) had previous cardiac surgery. Case 3 had a bicuspid valve.

E-OHS was necessary in seven cases of ventricular rupture (63%), two cases of coronary occlusion (18%), an aortic dissection associated at valve pop-up (9%) and a mitro-aortic junction rupture (9%).

The E-OHS in these patients has been fundamental in all cases of ventricle rupture (7/7 survived) and coronary occlusion (1/2 survived, 50%). We failed the E-OHS in those cases with serious consequences on the internal cardiac structures (massive mitral regurgitation after coronary occlusion, aortic dissection, mitro-aortic junction rupture) the cumulative mortality was 27% (3/11).

In the absence of a complete cardiac surgery rescue team, the deaths of these patients would presumably be close to 100%. Consequently, the rapid E-OHS saved eight patients, giving a decrease of 0.97% in our mortality.

After highlighting the increased incidence of ventricular ruptures, we investigated the material used and found an excessively stiff wire and therefore replaced it.

In all cases of E-OHS, the heart team strategy limited the use of surgery only to stabilize the hemodynamics and solve the surgical problems. In fact, after the resolution of the emergency, we always preferred to continue with the percutaneous approach (example cases 1–4 with the implantation of TAVI or cases 5–6 with coronary reperfusion obtained by PCI rescue supported by cardiopulmonary assistance) to keep the team roles. The only cases in which we performed a surgical strategy were in the aortic dissection and in the mitro-aortic junction rupture due to the impossibility of resolve the problem with percutaneous approach.

It has also been very important to always choose the less invasive strategy to solve problems without aortic cross-clamp (8/11 patients 72%) to minimize the damaging effects on these fragile patients (7/8 survived 87.5%). We performed aortic cross-clamp only in three (27%) patients (1/3 survived, 33.3%).

Another very important finding to note is that in all cases of E-OHS we did not have time to reach the surgical room and we had to face CA directly in the hemodynamics room. Therefore, is evident that the presence of a complete rescue team with cardiac surgeon, perfusionist and surgical nurse, trained to emergency management, and working in a high-volume cardiac centre on site is the real cause that reduce the time of rescue, and consequently the mortality, instead of the presence of any iOSCS.

## 4. Discussion

The use of TAVI has been expanded to intermediate- and low-risk patients thanks to the procedure’s safety, good results, and reduced invasiveness. The volume of TAVI has increased over the last decades.

Our results on 825 consecutive TAVI patients showed promising results with a low overall mortality rate (2.3%) despite the high average age and comorbidities of patients, confirming the already reported safety of the technique [3,4].

These good data are probably due to many factors: we had a high volume of heart valve surgery, expert interventional cardiology, and an expert cardiac surgeon performing TAVI together and ready for any emergencies.

Even if the incidence of acute so-called “E-OHS” open intervention after TAVI remains low (1–3%), this intervention is until associated with significantly increased morbidity and mortality.

Our study was also focused on the need for an E-OHS during the procedure. Evidently, the need for this approach is very rare, but it can save some lives that would otherwise be lost.

Nowadays, the cardiac surgeon back-up is still recommended to perform that procedure, but in recent years, it has been questioned. In the absence of on-site cardiac surgery, the two currently adopted options are a visiting surgical team for back-up during intervention or an external cardiac surgery facility on-site.

This need for cardiac surgeon back-up limits many interventional cardiologists, significantly lengthening waiting lists and mortality for patients waiting for surgery.

On the other hand, it is evident how the possible absence of the cardiac surgeon would leave interventional cardiologists alone in managing complications and increase waiting times for patients before being rescued.

Kobo et al. have already analyzed these problems. Their review estimated a mortality rate ranging from 2% to 6% for TAVI procedures in centers without iOSCS. At the same time, they evidenced the need for cardiac surgery in about 1% of total cases. Instead, the mortality rate for patients during waiting time for procedures ranged from 2% to 10–14% in some isolated areas like Ontario in Canada [22].

A few years ago, Pineda et al. reported data from the Society of Thoracic Surgeons/American College of Cardiology TVT Registry with 47,546 patients undergoing TAVI from 2011 to 2015 and a 1.2% rate of E-OHS. In these patients, the mortality rate was high (50% at 30 days) due to different causes [23]. Li et al. reported a mortality rate of 35% at thirty days [17], Fagu et al. reported an all-cause mortality of 50% [18], and Cuartas et al. reported a similar mortality rate (In-hospital mortality 56.8%) in E-OHS [15]. A recent article from Foglietta et al. evidenced that data from randomized controlled trials and registries failed to document any difference in outcomes and conversion rate to E-OHS in centers with or without iOSCS; on the other hand, a direct relationship with TAVI complications has been clearly documented for learning curve and center volume [16]. Our data show how the presence of a rescue team can make these procedures safer; we had a mortality rate at 30 days of 27% for patients who needed of E-OHS. This result is better than what has already been described by other authors. These promising results are probably related to the contemporary presence of both medical figures at the operating table ensuring faster rescue times. Moreover, the immediate availability of surgical instruments, surgical nurses, a perfusionist, and a CPB machine decreases the duration of hemodynamic instability or the time to resuscitate. It allows us to establish mechanical support and perform a sternotomy.

Must be highlighted the fact that we did not reach in all cases the surgery room and we performed E-OHS in hemodynamic room directly.

Without all these factors, the deaths of these patients would be nearly 100%. Therefore, we can deduce that more than half of these patients could be saved by immediate surgical conversion with circulatory support by cardiopulmonary bypass. This finding contradicts previous authors who say that cardiac surgeon with the complete rescue team is not necessary and is not a valid safety for patients having TAVI [24]. Choosing the less invasive way to solve the problem during the E-OHS, always trying to avoid aortic cross clamp and concluding the percutaneous procedure, resulted in a reduction of mortality. In fact, the mortality rate in patients with aortic cross clamping is significantly greater than the other group (66.6% vs. 12.5%; *p* < 0.005). Finally, from our experience, it is evident how the presence of a rescue team is still necessary and how that presence, thanks to the habit of managing emergencies (working in a high-volume cardiac center and generally trained to the management of emergencies), can improve results during possible TAVI complications. At the same time, we believe that during E-OHS, the heart team strategy should limit the use of surgery only to stabilize the hemodynamics and solve the active surgical problems. Indeed, also in emergent scenario after resolution of surgical problem, our policy is to continue with the percutaneous approach and always choose the less invasive strategy to solve complications without aortic cross-clamp to minimize the damaging effects on these fragile patients.

In all cases, it is fundamental to obtain the informed consent of patients. They obviously must be informed about all the risks of the procedure but also about the risks of an E-OHS and the team that should run it. Patients must have the possibility to choose the center and prefer one with an iOSCS rather than one with a visiting surgical team for back-up during intervention or an external cardiac surgery facility on-site.

Finally, only cardiac surgeons with expertise on a broad, complex spectrum of clinical scenarios and techniques to address open surgical interventions after transcatheter valve implantations can improve survival in these catastrophic events.

## 5. Limitations

There are limitations to the current study. Firstly, using a retrospective design introduces potential bias and limitations in data collection. Moreover, the absence of a control group hampers our ability to draw definitive conclusions regarding the effectiveness of the intervention. Additionally, the relatively small sample size and three-center setting raise concerns about the generalizability of the findings to a broader population.

Finally, the follow-up is limited to 30 days.

To address these limitations, future research should include larger prospective studies involving multiple centers.

## 6. Conclusions

TAVI remains a complicated procedure with rare complications requiring fast surgical treatment. The presence of a complete and skilled rescue team available during TAVI means an increase in survival for a high number of patients.

TAVI procedures should be performed in a high-volume center of cardiac valve surgery, with a well-trained multidisciplinary team and an immediate surgical back-up to increase patient safety and outcomes.

Although further studies are needed, this study leads to the final hypothesis that is not the structure, but the expert and complete team presence on site who is necessary to save most life as possible.

## Figures and Tables

**Table 1:** Baseline characteristics of 825 patients. SD: Standard Deviation; BMI: Body Mass Index; COPD: Chronic Obstructive Pulmonary Disease.

Carachteristic	Total Patients	E-OHS Patients (11)
Age Year Mean (SD)	79 Years (6.4)	78.6 Years (5.95)
Sex	429 males (52%)	4 males (36%)
	396 females (48%)	7 females (64%)
BMI (SD)	28.3 (5.1)	26.5 (4.3)
Coronary artery disease (%)	42/825 (5.1%)	0 (0%)
Atrial fibrillation (%)	143 (17.3%)	3 (27%)
Diabetes (%)	256 (31%)	6 (54%)
COPD (%)	99 (12%)	3 (27%)
Arterial hypertension (%)	552 (67%)	10 (91%)
Chronic kidney disease (%)	42 (5%)	2 (18%)
Aortic valve stenosis (%)	825 (100%)	11 (100%)
Urgency	779 elective (94.4%)	11 (100%)
	46 urgent (5.6%)	0 (0%)
EuroSCORE II expected (SD)	Average 7% (2)	4.9% (1.77)
Valve anatomy	779 tricuspid (94.4%)	8 (73%)
	46 bicuspid (5.6%)	3 (27%)
Previous cardiac surgery	149 (18%) 64 valve-in-valve (7.8%)	2 (18%) 0 (0%)
Frailty score (SD)	-	4.1 (0.7)

**Table 2:** Operative outcomes. PCI: Percutaneous Coronary Intervention.

	Results
Mortality (30 days)	19/825 (2.3%)
Cardiogenic shock	13 cases (1.6%) 11 E-OHS (1.3%) 2 pharmacological support (0.3%)
Survival after E-OHS at 30 days	73%
Heart perforation	8 (1%)
Vascular complications	10 major (1.2%) 26 minor (3.2%)
Contemporary PCI	42 (5.1%)
Definitive pacemaker implantation	58 (7%)
In-hospital stay (days)	10.45 ± 3
Type of prosthesis	448 Corevalve Evolute R (54.3%) 281 Portico (34.1%) 94 Corevale (11.4%) 2 Myval (0.2%)
Dimension of prosthesis	Average 27.5 mm 120 cases 26 mm (21%) 71 cases 27 mm (12.5%) 159 cases 29 mm (28%) 220 others (38.5%)
Approaches	Trans-femoral 813 (98.6%) Trans-subclavian 6 (0.7%) Trans-axillary 6 (0.7%)

**Table 3:** E-OHS case synthesis according to VARC 3 criteria. EF: Ejection Fraction.

	1	2	3	4	5	6	7	8	9	10	11	Tot/Mean
Age	85	84	70	65	82	76	78	82	82	78	83	78.6
Sex	M	M	F	F	F	M	F	M	F	F	F	4M/7F
EuroSCORE (%)	5.03	4.88	5.18	5.98	4.1	7.03	4.46	1.7	3.35	4.1	8.3	4.9
EF (%)	45	45	50	60	40	55	40	55	55	60	45	50
Mortality	NO	YES	NO	NO	YES	NO	NO	NO	YES	NO	NO	3/11
Neurologic events	NO	NO	NO	NO	NO	NO	NO	YES	NO	NO	YES	2/11
Hospitalization (Days)	18	5	11	9	0	30	15	7	1	7	20	
Bleeding and transfusions	YES	YES	NO	YES	YES	YES	YES	NO	YES	YES	YES	9/11
Vascular and access-related complications	NO	NO	NO	NO	NO	NO	NO	NO	NO	NO	NO	0/11
Cardiac structural complications	YES	YES	YES	YES	YES	YES	YES	YES	YES	YES	YES	11/11
Other procedural or valve-related complications	YES	YES	YES	YES	YES	YES	YES	YES	YES	YES	YES	11/11
New conduction disturbances and arrhythmias	NO	NO	YES	NO	NO	NO	NO	NO	NO	NO	YES	2/11
Acute kidney injury	NO	YES	NO	NO	NO	YES	NO	NO	YES	NO	YES	4/11
Myocardial infarction	NO	YES	NO	NO	YES	NO	NO	NO	YES	NO	NO	3/11
Bioprosthetic valve dysfunction	NO	NO	NO	NO	NO	NO	NO	NO	YES	NO	NO	1/11
Leaflet thickening and reduced motion	NO	NO	NO	NO	NO	NO	NO	NO	NO	NO	NO	0/11
Clinically significant valve thrombosis	NO	NO	NO	NO	NO	NO	NO	NO	NO	NO	NO	0/11
Aortic valve regurgitation	YES	NO	NO	NO	NO	YES	NO	NO	YES	NO	NO	3/11
Patient-reported outcomes and health status	FOLLOW-UP TOO SHORT TO EVALUATE											
Composite endpoints												
Technical success	NO	NO	NO	NO	NO	NO	NO	NO	NO	NO	NO	0/11
Device success	NO	NO	NO	NO	NO	NO	NO	NO	NO	NO	NO	0/11
Early safety	NO	NO	NO	NO	NO	NO	NO	NO	NO	NO	NO	0/11
Clinical efficacy	FOLLOW-UP TOO SHORT TO EVALUATE											

## Data Availability

Data are not published on publicly archive, but are available after mail request to the corresponding author.
